# Angiotensin II modulates CD40 expression in vascular smooth muscle cells

**DOI:** 10.1042/CS20080155_EOC

**Published:** 2026-09-10

**Authors:** Heraldo P. Souza, Denise Frediani, Ana L. Cobra, Ana I. Moretti, Márcia C. Jurado, Thadeu R. Fernandes, Arturo J. Cardounel, Jay L. Zweier, Rita C. Tostes

**Affiliations:** 1 Department of Emergency Medicine, School of Medicine, University of Sao Paulo, 01246-9013 Sao Paulo, Brazil; 2 Department of Pharmacology, Institute of Biomedical Sciences, University of Sao Paulo, 05508-900 Sao Paulo, Brazil; 3 Davis Heart & Lung Research Institute, Ohio State University, Columbus, OH 43210, U.S.A.

**Keywords:** atherosclerosis, CD40, cytokine, electron paramagnetic resonance, hypertension, superoxide

*Clinical Science* has been made aware of potential issues surrounding the scientific validity of this paper and is hence issuing a permanent Expression of Concern to notify readers. Some instances of high similarity were observed by a reader within Figure 5; specifically, between the Figure 5A and 5B GAPDH bands (lanes 1-4), and between the 5B CD40 mRNA bands lanes 3 and 4. Upon investigation, the Editorial Office noted additional similarities between the Figure 2 a-Actin/TNF-a bands (lanes 7-9) and the Figure 6 a-Actin/IL-1B and Angll bands (lanes 3-6), and between the Figure 2 a-Acin 4/5th lane and 6/7th lane bands.

The authors cooperated with the investigation but given the age of the article were unable to provide raw data or explain the similarities. Upon detailed analysis, the Figure 5A and 5B GAPDH bands do appear to have enough differences to alleviate concerns. For the remaining similarities, concerns remain. As such, the article will remain published but with an associated Expression of Concern.

